# Supplementary material for: Type 2 Diabetes-Related Variants Influence the Risk of Developing Prostate Cancer: A Population-Based Case-Control Study and Meta-Analysis
Source: Cancers (Basel). 2022 May 12;14(10):2376. doi: 10.3390/cancers14102376 (PMC9139180; doi:10.3390/cancers14102376)
Supplement: Supplementary file 1 [file cancers-14-02376-s001.zip › Sanchez-Maldonado JM [1]. Supplementary Table_S3_Annotation.pdf]

Supplementary Table S3. Summary of functional and regulatory annotation of most significant SNPs.

Gene	dbSNP	Chr	Position (hg38)	Ref. allele	Risk allele	PHM[a,b]	EHM[a,b]	eQTL[c]	Regulatory Motifs Changed[d]	Proteins Bound[d]
ADAM30	*ADAM30*$_{rs2641348}$	1	119895261	A	G	ESC.H9<br>ESDR.H1.BMP4.TROP<br>LIV.ADLT<br>BLD.DND41.CNCR	ESC.I3<br>BLD.CD34.CC<br>BLD.CD15.PC<br>SKIN.PEN.FRSK.FIB.01<br>SKIN.PEN.FRSK.FIB.02<br>SKIN.PEN.FRSK.KER.03<br>BRN.GRM.MTRX<br>OVRY<br>PLCNT.FET<br>LIV.ADLT<br>PANC<br>BLD.DND41.CNCR<br>LIV.HEPG2.CNCR<br>BRST.HMEC<br>SKIN.NHEK	$9.75 \bullet 10^{-06}$<br>RP5-1042I8.7<br>$1.38 \bullet 10^{-08}$<br>NOTCH2<br>0.0026<br>REG4	Cphx<br>Hoxb6<br>Nkx2_10<br>Nkx2_8	
FTO	*FTO*$_{rs9939609}$	16	53786615	T	A	ESC.WA7<br>BLD.CD8.NPC<br>FAT.MSC.DR.ADIP	ESDR.H1.MSC<br>BLD.CD4.CD25M.CD45RA.NPC<br>BLD.CD4.CD25M.IL17M.PL.TPC<br>BLD.CD8.NPC<br>STRM.MRW.MSC<br>SKIN.PEN.FRSK.KER.03<br>BRST.MYO<br>BRN.HIPP.MID<br>BRN.ANT.CAUD<br>BRN.CING.GYR<br>BRN.DL.PRFRNTL.CRTX<br>FAT.ADIP.NUC<br>MUS.SKLT.F<br>HRT.ATR.R<br>HRT.VENT.L<br>HRT.VNT.R<br>VAS.AOR<br>GI.CLN.SM.MUS<br>GI.STMC.FET<br>GI.S.INT<br>GI.ESO<br>GI.STMC.GAST<br>LNG.FET<br>OVRY<br>PANC.ISLT<br>LNG.A549.ETOH002.CNCR<br>BRST.HMEC<br>SKIN.NHEK		Nanog_disc4<br>Pou5f1_disc1<br>Pou5f1_known1	
HNF1B	*HNF1B*$_{rs7501939}$	17	37741165	T	C	ESC.HUES6	ESC.HUES48	$9.2 \bullet 10^{-04}$	CEBPB_know	

						ESC.HUES64<br>ESC.4STAR<br>IPSC.20B<br>IPSC.15b<br>ESDR.CD184.ENDO<br>ESDR.H1.MSC<br>BLD.CD4.CD25M.CD45RA.NPC<br>BLD.CD4.CD25M.IL17M.PL.TPC<br>BLD.CD4.CD25M.CD45RO.MPC<br>BLD.CD4.MPC<br>FAT.ADIP.DR.MSC<br>BRN.CING.GYR<br>FAT.ADIP.NUC<br>GI.CLN.SM.MUS<br>GI.RECT.SM.MUS<br>GI.L.INT.FET<br>GI.CLN.MUC<br>GI.RECT.MUC.29<br>GI.RECT.MUC.31<br>GI.STMC.MUC<br>GI.DUO.MUC<br>KID.FET<br>LNG.FET<br>PANC.ISLT<br>LIV.ADLT<br>LNG.A549.ETOH002.CNCR<br>LIV.HEPG2.CNCR	ESC.4STAR<br>IPSC.20B<br>IPSC.18<br>ESDR.CD184.ENDO<br>ESDR.H1.MSC<br>BLD.CD4.CD25.CD127M.TREGPC<br>BLD.CD4.CD25M.TPC<br>BLD.CD4.CD25M.CD45RA.NPC<br>BLD.CD4.CD25M.IL17M.PL.TPC<br>BLD.CD4.CD25M.IL17P.PL.TPC<br>BLD.CD4.CD25M.CD45RO.MPC<br>BLD.CD4.MPC<br>BLD.CD8.MPC<br>BLD.CD8.NPC<br>BRN.HIPP.MID<br>BRN.SUB.NIG<br>BRN.CING.GYR<br>GI.DUO.SM.MUS<br>GI.CLN.SM.MUS<br>GI.S.INT.FET<br>GI.L.INT.FET<br>GI.CLN.MUC<br>GI.RECT.MUC.29<br>GI.RECT.MUC.31<br>GI.STMC.MUC<br>GI.DUO.MUC<br>PANC.ISLT<br>LIV.ADLT<br>LNG.A549.ETOH002.CNCR<br>LIV.HEPG2.CNCR	HNF1B	n3<br>DMRT5<br>DMRT7<br>p300_disc2	
HNF1B	*HNF1B*rs757210	17	37736525	T	C	ESDR.H1.NEUR.PROG<br>LIV.ADLT	ESC.4STAR<br>IPSC.DF.19.11<br>ESDR.CD184.ENDO<br>ESDR.H1.MSC<br>BRST.HMEC.35<br>GI.CLN.MUC<br>PLCNT.AMN<br>PANC<br>LNG.A549.ETOH002.CNCR	0.00177<br>HNF1B	HES1	
HNF1B	*HNF1B*rs4430796	17	37738049	G	A	IPSC.15b<br>KID.FET<br>LIV.ADLT<br>LNG.A549.ETOH002.CNCR	LNG.IMR90<br>ESC.WA7<br>ESC.I3<br>ESC.HUES48<br>ESDR.CD56.MESO<br>ESDR.CD184.ENDO<br>ESDR.H1.BMP4.MESO<br>ESDR.H1.MSC<br>STRM.MRW.MSC<br>STRM.CHON.MRW.DR.MSC<br>FAT.ADIP.DR.MSC	0.0024<br>HNF1B	Maf_disc2	STAT3

							FAT.MSC.DR.ADIP<br>SKIN.PEN.FRSK.KER.03<br>BRST.HMEC.35<br>GI.S.INT.FET<br>GI.L.INT.FET<br>GI.CLN.MUC<br>GI.STMC.MUC<br>GI.STMC.GAST<br>PLCNT.AMN<br>PLCNT.FET<br>LIV.ADLT<br>LNG.A549.ETOH002.CNCR<br>MUS.HSMMT<br>BRN.NHA<br>BONE.OSTEO			
JAZF1	*JAZF1*$_{rs10486567}$	7	27936944	G	A	LNG.A549.ETOH002.CNCR<br>FAT.ADIP.DR.MSC	LNG.IMR90<br>ESDR.CD184.ENDO<br>STRM.MRW.MSC<br>STRM.CHON.MRW.DR.MSC<br>FAT.ADIP.DR.MSC<br>FAT.MSC.DR.ADIP<br>SKIN.PEN.FRSK.FIB.01<br>SKIN.PEN.FRSK.FIB.02<br>SKIN.PEN.FRSK.MEL.01<br>SKIN.PEN.FRSK.MEL.03<br>BRN.HIPP.MID<br>BRN.SUB.NIG<br>BRN.CING.GYR<br>BRN.INF.TMP<br>BRN.DL.PRFRNTL.CRTX<br>FAT.ADIP.NUC<br>MUS.PSOAS<br>MUS.SKLT.F<br>MUS.SKLT.M<br>MUS.TRNK.FET<br>MUS.LEG.FET<br>HRT.FET<br>HRT.ATR.R<br>GI.CLN.SM.MUS<br>GI.RECT.SM.MUS<br>GI.CLN.SIG<br>GI.RECT.MUC.29<br>KID.FET<br>LNG.FET<br>PANC.ISLT<br>LNG.A549.ETOH002.CNCR<br>MUS.HSMMT<br>SKIN.NHDFAD<br>BONE.OSTEO		Ik-2_2<br>Lhx3_2<br>Mef2_known1<br>Mef2_known4<br>Nkx3_2<br>Zfp410	p300
LTA	*LTA*$_{rs1041981}$	6	31573007	C	A	ESDR.H1.MSC	IPSC.DF.19.11	$2.05 \bullet 10^{-09}$	LBP-1_3	

						BLD.PER.MONUC.PC<br>BLD.CD3.PPC<br>BLD.CD4.CD25I.CD127.TMEMPC<br>BLD.CD3.CPC<br>BLD.CD4.CD25.CD127M.TREGP C<br>BLD.CD4.CD25M.TPC<br>BLD.CD4.CD25M.CD45RA.NPC<br>BLD.CD4.CD25M.IL17M.PL.TPC<br>BLD.CD4.CD25M.IL17P.PL.TPC<br>BLD.CD4.CD25M.CD45RO.MPC<br>BLD.CD4.MPC<br>BLD.CD8.MPC<br>BLD.CD4.NPC<br>BLD.CD8.NPC<br>BLD.CD19.CPC<br>BLD.CD19.PPC<br>BLD.CD56.PC<br>SKIN.PEN.FRSK.FIB.01<br>SKIN.PEN.FRSK.FIB.02<br>SKIN.PEN.FRSK.FIB.02<br>BRST.MYO<br>THYM<br>THYM.FET<br>BRN.INF.TMP<br>BRN.FET.M<br>MUS.SKLT.F<br>HRT.FET<br>GI.RECT.SM.MUS<br>GI.STMC.MUS<br>GI.S.INT<br>GI.CLN.MUC<br>GI.RECT.MUC.29<br>GI.STMC.MUC<br>GI.DUO.MUC<br>GI.ESO<br>PLCNT.AMN<br>LIV.ADLT<br>BLD.GM12878<br>CRVX.HELAS3.CNCR<br>LIV.HEPG2.CNCR<br>MUS.HSMMT	ESDR.CD56.MESO<br>ESDR.H1.BMP4.TROP<br>ESDR.H1.MSC<br>BLD.PER.MONUC.PC<br>BLD.CD3.PPC<br>BLD.CD4.CD25I.CD127.TMEMPC<br>BLD.CD3.CPC<br>BLD.CD4.CD25.CD127M.TREGPC<br>BLD.CD4.CD25M.TPC<br>BLD.CD4.MPC<br>BLD.CD8.MPC<br>BLD.CD4.NPC<br>BLD.CD8.NPC<br>BLD.CD14.PC<br>BLD.CD19.CPC<br>BLD.CD34.PC<br>BLD.MOB.CD34.PC.M<br>BLD.MOB.CD34.PC.F<br>BLD.CD34.CC<br>BLD.CD19.PPC<br>BLD.CD56.PC<br>SKIN.PEN.FRSK.FIB.01<br>SKIN.PEN.FRSK.FIB.02<br>THYM<br>THYM.FET<br>MUS.SKLT.M<br>HRT.VNT.R<br>GI.STMC.MUS<br>GI.S.INT<br>GI.CLN.MUC<br>GI.RECT.MUC.29<br>GI.DUO.MUC<br>GI.ESO<br>PLCNT.AMN<br>LIV.ADLT<br>LNG<br>SPLN<br>BLD.GM12878<br>MUS.HSMMT<br>BLD.CD14.MONO	PSMB9<br>$3.09\bullet 10^{-09}$<br>YIF1B<br>$8.57\bullet 10^{-07}$<br>HLA-DRB5<br>$3.44\bullet 10^{-06}$<br>CYP21A1P<br>$3.47\bullet 10^{-06}$<br>LY6G6C<br>$1.78\bullet 10^{-07}$<br>HCG22<br>0.0017<br>AIF1<br>$2.636\bullet 10^{-16}$<br>BAT1<br>$1.73\bullet 10^{-04}$<br>CSNK2B<br>$3.53\bullet 10^{-04}$<br>HCP5<br>$5.15\bullet 10^{-05}$<br>LST1<br>$7.50\bullet 10^{-12}$<br>TNF	RFX5_disc1<br>Sin3Ak-20_disc6<br>Znf143_disc3	
MADD	*MADD*$_{rs7944584}$	11	47314769	A	T	ESC.WA7<br>BLD.CD4.NPC<br>FAT.ADIP.DR.MSC<br>SKIN.PEN.FRSK.FIB.01<br>SKIN.NHDFAD	LNG.IMR90<br>BLD.MOB.CD34.PC.F<br>FAT.ADIP.DR.MSC<br>SKIN.PEN.FRSK.FIB.01<br>FAT.ADIP.NUC<br>HRT.ATR.R<br>MUS.HSMMT<br>SKIN.NHDFAD	$5.12\bullet 10^{-06}$<br>NUP160<br>$2.42\bullet 10^{-06}$<br>SPI1<br>$9.36\bullet 10^{-06}$<br>ACP2<br>0.0022<br>CUGBP1	AP-1_known1<br>AP-1_known2<br>AP-1_known3<br>AP-1_known4<br>AP-4_1<br>BATF_disc1<br>Irf_disc2<br>KAP1_disc1	

							LNG.NHLF<br>BONE.OSTEO	$1.29 \bullet 10^{-07}$<br>MADD<br>$1.954 \bullet 10^{-14}$<br>MYBPC3|MADD	NF-E2_known1	
NOTCH	*NOTCH*$_{rs10923931}$	1	119975336	G	T	ESC.WA7<br>ESDR.CD184.ENDO<br>BLD.CD15.PC<br>FAT.MSC.DR.ADIP<br>FAT.ADIP.NUC<br>BLD.CD14.MONO	BLD.CD14.PC<br>BLD.MOB.CD34.PC.M<br>BLD.MOB.CD34.PC.F<br>BLD.CD34.CC<br>BLD.CD15.PC<br>STRM.CHON.MRW.DR.MSC<br>FAT.ADIP.DR.MSC<br>FAT.MSC.DR.ADIP<br>MUS.SAT<br>SKIN.PEN.FRSK.FIB.01<br>SKIN.PEN.FRSK.FIB.02<br>SKIN.PEN.FRSK.MEL.03<br>SKIN.PEN.FRSK.KER.02<br>SKIN.PEN.FRSK.KER.03<br>BRST.HMEC.35<br>BRST.MYO<br>BRN.CRTX.DR.NRSPHR<br>THYM<br>BRN.HIPP.MID<br>BRN.SUB.NIG<br>BRN.ANT.CAUD<br>BRN.ANG.GYR<br>BRN.DL.PRFRNTL.CRTX<br>FAT.ADIP.NUC<br>MUS.PSOAS<br>MUS.SKLT.F<br>HRT.ATR.R<br>VAS.AOR<br>GI.CLN.SM.MUS<br>GI.RECT.SM.MUS<br>BLD.CD14.MONO<br>GI.CLN.SIG<br>GI.RECT.MUC.29<br>GI.ESO<br>GI.STMC.GAST<br>LIV.ADLT<br>LNG<br>SPLN<br>BRST.HMEC<br>MUS.HSMMT<br>SKIN.NHDFAD<br>SKIN.NHEK<br>BONE.OSTEO	$2.7 \bullet 10^{-03}$<br>REG4<br>$7.3 \bullet 10^{-06}$<br>NOTCH		
RBMS1	*RBMS1*$_{rs7593730}$	2	160314943	T	C	STRM.CHON.MRW.DR.MSC	LNG.IMR90	$3.31 \bullet 10^{-07}$		

						FAT.ADIP.DR.MSC<br>FAT.MSC.DR.ADIP<br>FAT.ADIP.NUC<br>MUS.SKLT.F<br>MUS.SKLT.M<br>GI.CLN.SM.MUS<br>GI.RECT.MUC.29<br>MUS.HSMM<br>MUS.HSMMT<br>SKIN.NHDFAD	ESC.WA7<br>ESC.H9<br>LNG.IMR90<br>ESC.WA7<br>ESC.H9<br>ESC.I3<br>ESC.HUES64<br>ESC.4STAR<br>IPSC.20B<br>ESDR.H9.NEUR.PROG<br>ESDR.H9.NEUR<br>ESDR.CD56.MESO<br>ESDR.H1.BMP4.TROP<br>BLD.CD4.CD25I.CD127.TMEMPC<br>BLD.CD4.CD25M.TPC<br>BLD.CD4.CD25M.CD45RA.NPC<br>BLD.CD4.CD25M.IL17M.PL.TPC<br>BLD.CD4.CD25M.IL17P.PL.TPC<br>BLD.CD4.CD25M.CD45RO.MPC<br>BLD.CD4.MPC<br>BLD.CD8.MPC<br>BLD.CD4.NPC<br>BLD.CD8.NPC<br>STRM.MRW.MSC<br>STRM.CHON.MRW.DR.MSC<br>FAT.ADIP.DR.MSC<br>FAT.MSC.DR.ADIP<br>MUS.SAT<br>SKIN.PEN.FRSK.FIB.01<br>SKIN.PEN.FRSK.FIB.02<br>SKIN.PEN.FRSK.MEL.01<br>SKIN.PEN.FRSK.MEL.03<br>SKIN.PEN.FRSK.KER.03<br>BRST.MYO<br>BRN.HIPP.MID<br>BRN.ANT.CAUD<br>BRN.DL.PRFRNTL.CRTX<br>FAT.ADIP.NUC<br>MUS.PSOAS<br>MUS.SKLT.F<br>MUS.SKLT.M<br>MUS.TRNK.FET<br>HRT.FET<br>HRT.VENT.L<br>HRT.VNT.R<br>GI.DUO.SM.MUS<br>GI.CLN.SM.MUS<br>GI.RECT.SM.MUS<br>GI.S.INT<br>GI.RECT.MUC.31	RBMS1<br>$1.47 \bullet 10^{-05}$<br>ITGB6		

							GI.STMC.GAST<br>LNG.FET<br>OVRY<br>LIV.ADLT<br>SPLN<br>LNG.A549.ETOH002.CNCR<br>MUS.HSMM<br>MUS.HSMMT<br>VAS.HUVEC<br>BLD.CD14.MONO<br>BRN.NHA<br>SKIN.NHDFAD<br>LNG.NHLF<br>BONE.OSTEO			
SLC2A2	*SLC2A2*$_{rs11920090}$	3	170999732	T	A	ESC.H9<br>ESDR.CD184.ENDO<br>FAT.ADIP.DR.MSC<br>BRN.ANT.CAUD<br>GI.CLN.SM.MUS<br>GI.DUO.MUC<br>LIV.ADLT<br>LNG.A549.ETOH002.CNCR<br>LIV.HEPG2.CNCR<br>VAS.HUVEC<br>BRN.NHA<br>SKIN.NHEK	LNG.IMR90<br>ESC.HUES48<br>ESC.HUES64<br>ESDR.H9.NEUR.PROG<br>ESDR.H9.NEUR<br>ESDR.CD56.MESO<br>ESDR.CD184.ENDO<br>ESDR.H1.BMP4.MESO<br>ESDR.H1.MSC<br>ESDR.H1.BMP4.MESO<br>ESDR.H1.MSC<br>BLD.CD34.PC<br>BLD.MOB.CD34.PC.M<br>BLD.MOB.CD34.PC.F<br>BLD.CD34.CC<br>STRM.MRW.MSC<br>STRM.CHON.MRW.DR.MSC<br>FAT.ADIP.DR.MSC<br>FAT.MSC.DR.ADIP<br>MUS.SAT<br>SKIN.PEN.FRSK.FIB.01<br>SKIN.PEN.FRSK.FIB.02<br>SKIN.PEN.FRSK.MEL.03<br>SKIN.PEN.FRSK.KER.02<br>SKIN.PEN.FRSK.KER.03<br>BRST.HMEC.35<br>BRST.MYO<br>BRN.CRTX.DR.NRSPHR<br>BRN.HIPP.MID<br>BRN.SUB.NIG<br>BRN.ANT.CAUD<br>BRN.CING.GYR<br>BRN.ANG.GYR<br>FAT.ADIP.NUC<br>VAS.AOR<br>GI.DUO.SM.MUS		Prrx2_1	

							GI.CLN.SM.MUS<br>GI.RECT.SM.MUS<br>GI.STMC.MUS<br>GI.S.INT.FET<br>GI.L.INT.FET<br>GI.S.INT<br>GI.CLN.SIG<br>GI.RECT.MUC.29<br>GI.RECT.MUC.31<br>GI.STMC.MUC<br>GI.DUO.MUC<br>PLCNT.AMN<br>KID.FET<br>LNG.FET<br>PANC.ISLT<br>ADRL.GLND.FET<br>LIV.ADLT<br>PANC<br>LNG.A549.ETOH002.CNCR<br>CRVX.HELAS3.CNCR<br>LIV.HEPG2.CNCR<br>BRST.HMEC<br>MUS.HSMM<br>VAS.HUVEC<br>BLD.K562.CNCR<br>BRN.NHA<br>SKIN.NHDFAD<br>SKIN.NHEK<br>LNG.NHLF<br>BONE.OSTEO			
THADA	*THADA*$_{rs7578597}$	2	43505684	T	C	IPSC.18<br>BRN.DL.PRFRNTL.CRTX<br>OVRY	ESC.HUES6<br>ESC.HUES48<br>ESC.HUES64<br>IPSC.18<br>IPSC.15b<br>ESDR.CD56.ECTO<br>ESDR.CD184.ENDO<br>SKIN.PEN.FRSK.FIB.02<br>SKIN.PEN.FRSK.KER.03<br>BRN.GANGEM.DR.NRSPHR<br>BRN.SUB.NIG<br>LNG.FET<br>BLD.CD14.MONO		Foxo_3<br>Mef2_disc1<br>Pou5f1_disc1<br>Sox_10<br>Sox_13<br>Sox_15<br>Sox_16<br>Sox_18<br>Sox_19<br>Sox_2<br>Sox_4<br>Sox_5<br>Sox_9<br>Zfp105	

Abbreviations: PHM, protomer histone marks; EHM, enhancer histone marks; Ref, reference.
[a] HaploReg v4 based epigenomic changes
[b] CHc15sm, ChromHMM (core 15-state model); CHc25sm, ChromHMM (core 25-state model using 12 imputed marks); H3K4me1/H3K4me3, H3K4me1 and H3K4me3 peaks; H3K27ac/H3K9ac, H3K27ac and H3K9ac peaks

[c] eQTL data from Haploreg v4 (Whole blood).
[d] Functional annotation from the ENCODE based tool HaploReg v4 (website http://www.broadinstitute.org/mammals/haploreg/haploreg_v4.php)
